# Why some sites are responding better to anti-malarial interventions? A case study from western Kenya

**DOI:** 10.1186/s12936-017-2145-9

**Published:** 2017-12-29

**Authors:** Anthony Kapesa, Eliningaya J. Kweka, Harrysone Atieli, Erasmus Kamugisha, Guofa Zhou, Andrew K. Githeko, Guiyun Yan

**Affiliations:** 1 Climate and Health Laboratory, Centre for Global Health Research, Kenya Medical Research Institute, P.O. Box 1578, Kisumu, Kenya; 2 Catholic University of Health and Allied Sciences, P.O. Box 1464, Mwanza, Tanzania; 3 Division of Livestock and Human Health Disease Vector, Tropical Pesticides Research Institute, P.O. Box 3024, Arusha, Tanzania; 4 Program in Public Health, University of California, Irvine, CA 92697 USA

**Keywords:** Malaria intervention, Species shift, Climate variability, Insecticide resistance, Western Kenya

## Abstract

**Background:**

In sub-Saharan Africa, malaria interventions over the last decades have been successful in reducing both mortality and morbidity. In western Kenya however some areas experience contrasting outcomes of the ongoing interventions while the causes for this observation remains not yet clearly known.

**Methods:**

The WHO insecticide (deltamethrin) susceptibility test of the common malaria vectors was studied. Multiple surveys on household use and hospital prescriptions of antimalarial drugs from 2003 to 2015 were done. Along with this, cross sectional surveys on their availability in the local drug dispensing outlets were also done in 2015. Monthly precipitations and air temperature data was collected along with systematic review on abundance and composition of common malaria vectors in the study area before and during interventions. The above factors were used to explain the possible causes of contrasting outcome of malaria interventions between the three study sites.

**Results:**

Areas with malaria resurgence or sustained high transmission (Kombewa and Marani) showed higher composition of *Anopheles funestus* sensu lato (s.l.) than the previously abundant *Anopheles gambiae* sensu stricto (s.s.) and the later had higher composition to an area with a sustained infection decline (Iguhu). *Anopheles gambiae* s.l. from Kombewa showed highest resistance (50% mortality) upon exposure to WHO deltamethrin discriminating dosage of 0.75% while those from Marani and Iguhu had reduced resistance status (both had a mean mortality of 91%). Sampled *An. funestus* s.l. from Marani were also highly resistant to deltamethrin as 57% of the exposed vectors survived. An increasing of mean air temperature by 2 °C was noted for Marani and Iguhu from 2013 to 2015 and was accompanied by an increased rainfall at Marani. Community drug use and availability in selling outlets along with prescription in hospitals were not linked to the struggling control of the disease.

**Conclusions:**

The malaria vector species composition shift, insecticide resistance and climatic warming were the likely cause of the contrasting outcome of malaria intervention in western Kenya. Surveillance of malaria parasite and vector dynamics along with insecticide resistance and vector biting behaviour monitoring are highly recommended in these areas.

## Background

Malaria has been the major public health concern in many tropical and subtropical countries but the interventions have greatly reduced both morbidity and mortality cases [1]. Despite of the observed disease burden reduction, sub-Saharan still bears 90% of all malaria cases and 92% of mortalities [1]. Asymptomatic infections in Africa have been halved and clinical incidence of the disease reduced by 40% between 2000 and 2015 [2]. Similarly, malaria outpatient consultations in Kenya have dropped from 25 to 35% to 18% and from 20 to 6% of all hospital admissions [3, 4]. The increased access, ownership and use of the long-lasting insecticidal nets (LLINs) have greatly contributed to the decrease of morbidity [2]. The use of indoor residual insecticide spray (IRS) in combination with LLINs resulted to the observed reduction of disease burden [5, 6]. The LLINs ownership and use in Kenya have been consistently increasing since year 2000 [7, 8]. However, the massive application of IRS in western Kenya started in 2005 and by 2010 only 38% of households in epidemic prone areas were covered and even less in the recent years [4, 8]. Prompt diagnosis and management of malaria using efficacious anti-malarial drugs of choice remains one of the three important interventions [9–11]. The adoption of artemisinin-based combination therapy (ACT) recommended use of artemether 20 mg–lumefantrine 120 mg as first-line treatment for uncomplicated malaria since 2004 [12]. About 92.8% of children with fever in 2015 were given ACT and only 1.4% used sulfadoxine–pyrimethamine (SP) [7].

Despite of the interventions described above, some areas in western Kenya successfully controlled the disease while others experienced changing dynamics [13]. Similar observation of sustained high transmission despite of the available interventions has been also observed in other parts of sub-Saharan Africa in recent years [14, 15]. However, reasons contributing to this observation remain not clearly known in the midlist of the reported increasing vectors insecticide resistance and shift of vector populations in western Kenya [16, 17]. The known factors attributing to malaria resurgence and changing transmission dynamics [18] were used to explore the reasons contributing to the contrasting outcome of the available malaria interventions in western Kenya.

## Methods

### Study area

**Fig. 1:**
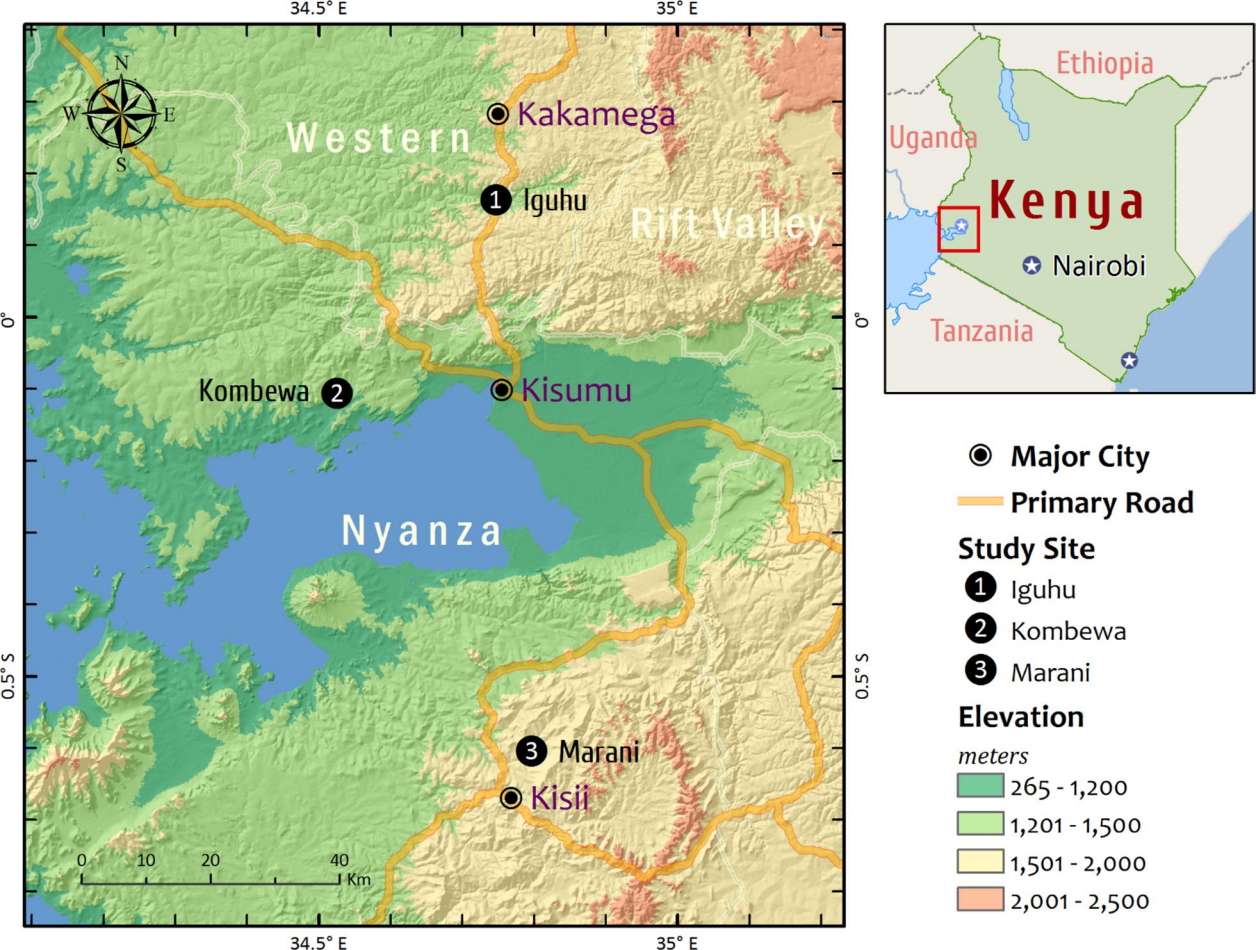
Map showing the three study sites with different intensity of malaria transmission; Iguhu (mesoendemic), Kombewa (endemic) and Marani (epidemic)

This study was conducted from three areas with different malaria transmission intensity namely; Marani in Kisii County (hypoendemic), Iguhu which is located in Kakamega County (mesoendemic) and Kombewa in Kisumu County which is in malaria hyperendemic zone (Fig. 1). The prevalence of asymptomatic malaria (2002–2010) among school age children for Kombewa, Iguhu and Marani were 47.1, 28.4 and 6.2% respectively [19]. Demographic characteristics of the population, topography, and climate together with malaria entomological information are described elsewhere [19].

### Parasitological and entomological surveillance

Monthly malaria finger pricks were done among school children and Giemsa-stained blood slide examined by microscope as from 2002 to 2016. Along with this, monthly pyrethroids spray catch to 30 randomly selected houses per site was also done. Total number of malaria confirmed cases were obtained from the outpatient register books of the three hospitals located in the study area from 2005 to 2016. The number of confirmed malaria cases were obtained from the local health facilities.

### Literature review on the composition of malaria vectors and interventions

Review of published research articles from the three study sites was done ranging from year 2000 to 2016. Electronic search of literatures was based on vector composition and abundance; biting activity; long lasting insecticide treated nets (LLINs) household ownership and use; indoor residual spray. The search was made with reference to the study sites on PubMed library. “*Malaria vector species composition and abundance in western Kenya*”; “*indoor residual spray coverage in western Kenya”* and *“long lasting insecticides treated mosquito nets coverage in western Kenya*”; “*Malaria vectors biting behavior in western Kenya”*. Furthermore, assessment of species composition and abundance of *Anopheles gambiae* complex in the study areas was done.

#### Malaria vectors insecticides susceptibility test

Insecticide susceptibility test was done using deltamethrin discriminating dosage (0.75%) for *Anopheles gambiae* sensu lato (s.l.) adult female mosquitoes using WHO tube bioassay guideline [20].

### Drug use, availability and prescription surveys

Multiple cross-sectional surveys of anti-malarial drug prescriptions in the outpatient department were done in year 2003, 2007, 2010 and 2015 in three hospitals located in the study area. Along with hospital surveys, community surveys on household anti-malarial drug use were also done in the same periods. A survey on availability of different types of anti-malarial drugs in community based drug outlets was done among 57 registered drug dispensing outlets in the study area.

### Review of precipitation and air temperature data

Retrospective review of monthly mean maximum, average and minimum rainfall and air temperature data from 2002 to 2015 obtained from nearby metrological stations.

### Data management

Out of 183 searched publications on “malaria vectors in western Kenya” 14 papers qualified for the review. The search on “LLINS and IRS coverage in western Kenya” found 36 research articles and 13 of them were selected. Four articles on vector biting behaviour were retrieved from the specific study sites. Mortality score of malaria vectors exposed to deltamethrin was done according to WHO guidelines and resistance was determined at the 90% mortality threshold. Comparison of anti-malarial drug use and availability was done by using Pearson Chi square or exact test. Graphic presentation on variability from the mean of the long-term precipitations and air temperature data was given per study site.

## Results

### Trend of asymptomatic and clinical malaria and populations of indoor resting malaria vectors

**Fig. 2:**
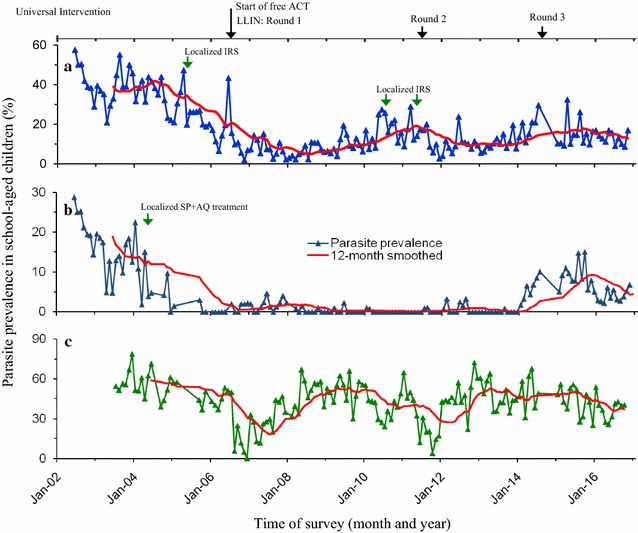
Long term trend of malaria parasitaemia in school age children in western Kenya. **a** Iguhu, **b** Marani, **c** Kombewa

**Fig. 3:**
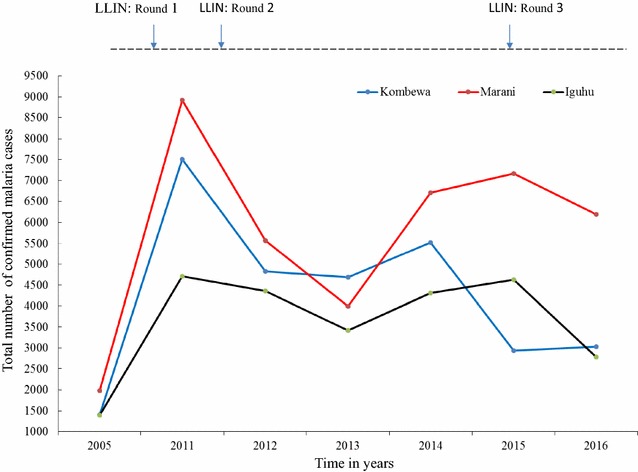
Trend of confirmed malaria cases in three health facilities located in different malaria transmission intensity in western Kenya from 2005 to 2016. (The red line includes both confirmed and clinical cases)

**Fig. 4:**
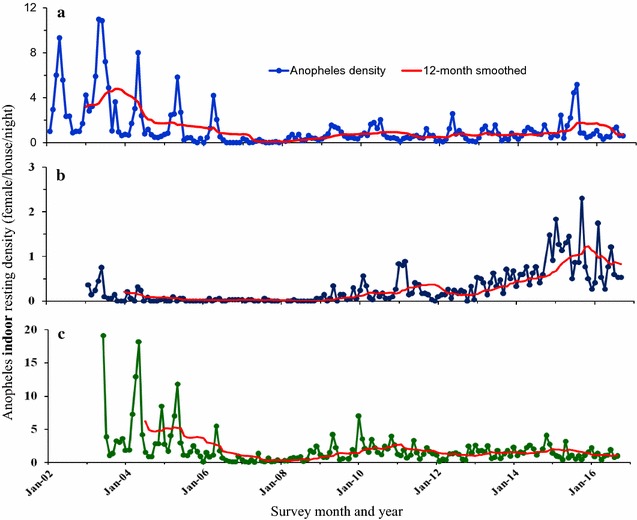
The trend of indoor resting malaria vectors density from three study sites in western Kenya. **a** Iguhu, **b** Marani, **c** Kombewa

The first cycle (2006) of LLINs mass distribution in western Kenya resulted to a decline of both asymptomatic malaria and clinical cases. The second cycle (2011) responded appropriately at Iguhu but Marani and Kombewa experienced resurgence and persistently high transmission respectively despite of the third round (2015) LLINs distributions (Figs. 2, 3). The population of indoor resting malaria vectors per house per night at Marani also increased from 0.03 in December 2007 to 1.07 in December 2016 Marani (Fig. 4). Whereas, vector composition at Kombewa and Iguhu remained with minor variations as from 2007 to 2016 but with consistently higher densities at Kombewa (Fig. 4).

### Composition of malaria vectors in areas with different responses of malaria interventions in western Kenya

**Fig. 5:**
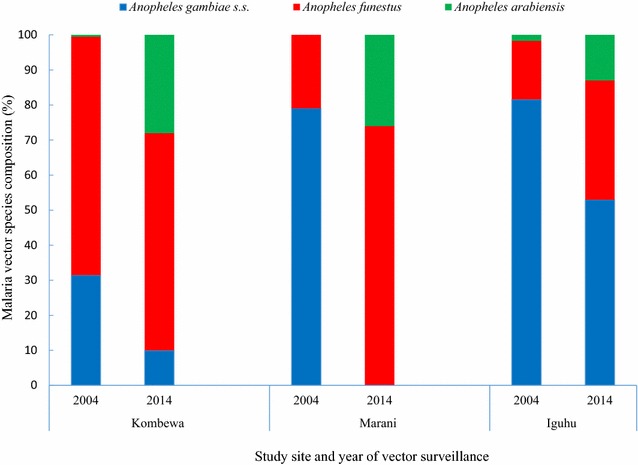
Species composition of malaria vectors before and during intensive interventions in three study sites with different responses to interventions in western Kenya

Studies from 2002 to 2004 (Iguhu and Marani) found 80% of all indoor resting vectors were *An. gambiae* sensu stricto (s.s.) and the rest were *Anopheles funestus* s.l. The vector density of *An. gambiae* s.s. was 4.95 and 0.19 per house per night for Iguhu and Marani respectively [21–23]. Meanwhile, *An. funestus* s.l. constituted about 68% of all indoor collected vectors at Kombewa with the mean density of 6.96 vectors per house per night [21, 24]. However, following increased LLINs coverage (January 2012 to June 2014), the population density of *An. gambiae* s.l. at Marani reduced from 0.19 to 0.06 vectors per house per night and that of *An. funestus* s.l. changed slightly from 0.19 to 0.17 [17, 21] (figure). Moreover, *An. funestus* s.l. (in 2014) became the main vector at Marani with a composition of 74% of all indoor collected malaria vectors. Between 2014 and 2016, the population of indoor resting vectors (mainly *An. funestus* s.l.) rose sharply (Fig. 4). Whereas at Kombewa, the density of *An. funestus* s.l. decreased from 6.96 in 2004 to 1.08 in 2014 and still constituted 62% of all indoor collected vectors. *An. gambiae* s.s. density decreased from 3.24 (2004) to 0.16 (2014) vectors per house per night and therefore making the composition drop from 32% to 10%. Iguhu similarly experienced decline of *An. gambiae* s.s. from 4.95 to 0.35 vectors per house per night, but still remained as the main vectors by 53% composition [17, 21] (Fig. 5). The decline of malaria vectors was also along with reduction of sporozoites rates at Iguhu while remaining constant at Kombewa and increased at Marani [17].

### Insecticide-treated mosquito nets (LLINs) household ownership and indoor residual spray (IRS) scale up in western Kenya

Using free mass distribution of the LLINs strategy started in 2006, the coverage and ownerships greatly improved [7, 13, 17]. Marani experienced a sharp increase in LLINs ownership from 11.8% in 2004 to 65.8% in 2008 and up to 80% in 2014 where as at Iguhu the coverage gradually increased from 12.8% in 2004 to 24.6% in 2007 before sharply rose to 78% in 2014. Conversely, the LLINs ownership was relatively higher at Kombewa in 2004 (52.3%) which then rose to 77.9% in 2010 and then over 80% in 2014 [13, 17, 19, 25] (Table 1). In western Kenya IRS started in 2005 and by 2010 only 38% of households in epidemic prone areas were sprayed and even less coverage in the recent years [4, 8]. From 2013 to 2015 IRS programme shifted only to few districts and this was due to some constraints including the emergence of resistance to the previously sprayed pyrethroids but also none of the study sites sprayed recent years [26].

**Table 1:** Coverage of long-lasting insecticidal nets (LLINs) from 2004 to 2015 from the three study sites showing different response of interventions in western Kenya

Year	Household LLINs ownership			References
	Kombewa (%)	Marani (%)	Iguhu (%)	
2004	52.3	11.8	12.8	[19]
2007/2008	65	65.8	24.6	[19]
2014	> 80	> 80	78	[17]
2015	> 80	> 80	> 80	[13]

### Susceptibility of *Anopheles gambiae* s.l. to deltamethrin in western Kenya

**Fig. 6:**
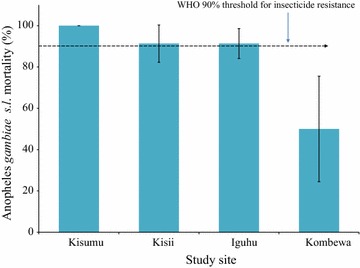
Mortality of *Anopheles gambiae* s.l. exposed to deltamethrin WHO bioassay from three study sites with different responses to malaria interventions in western Kenya

A total of 476 female *An. gambiae* s.l. were exposed to deltamethrin and 394 (82.7%) died after 24 h observation. Kombewa showed the highest deltamethrin resistance with the mean mortality of 50% out of 113 exposed female mosquitoes. *An. Gambiae* s.l. form Marani and Iguhu had reduced resistance as 91.3% died out of 175 and 166 of the exposed vectors respectively (Fig. 6). Moreover, *An. funestus* s.l. from Marani were resistant to deltamethrin as only 43% (21/37) of exposed female vectors died.

### Historical and current household drug use surveys

**Fig. 7:**
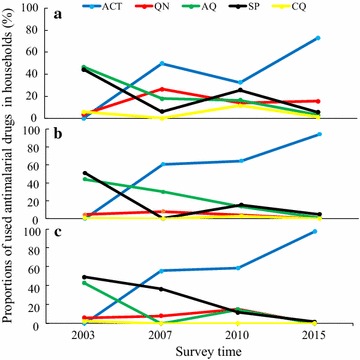
Trend of household anti-malarial drug use in three study sites of western Kenya from 2003 to 2015. **a** Iguhu, **b** Marani, **c** Kombewa

Before the introduction of ACT in western Kenya (2003) about 47% (487/1031), 44.9% (456/1031) and 4% (43/1031) of residents used amodiaquine, sulfadoxine–pyrimethamine and chloroquine, respectively, while only 4% (45/1031) reported using quinine. The proportion of ACT use in western Kenya was highest by 2015 (89% (1330/1493) while only 4.01% (60/1493), 1.2% (19/1493) and 0.4% (6/1493) of the residents treated malaria using SP, amodiaquine and chloroquine, respectively (Fig. 7). The household survey found more use of non-ACT oral drugs at Iguhu (9.6% (46/500) than Marani (6% (30/500) and Kombewa (1.6% (8/500) [χ^2^ = 27.54; p < 0.001] (Table 2). The study site with good response to malaria interventions showed more use of the already resistant anti-malarial drugs than those experiencing infection resurgence.

**Table 2:** Compared household use of different anti-malarial drugs from 2003 to 2015 of three study sites of western Kenya

Survey year	Study site	Type of anti-malarial drug used		χ^2^	*p* value
		(SP, AQ, CQ)	(ACT, QN)		
2015	Marani	6% (30/500)	94% (470/500)	27.54	< *0.0001*
	Kombewa	1.6% (8/500)	98.4% (492/500)		
	Iguhu	9.2% (46/500)	90.8% (454/500)		
2010	Marani	31.9% (91/285)	68.1% (194/285)	50.48	< *0.0001*
	Kombewa	26.1% (52/199)	73.9% (147/199)		
	Iguhu	53.6% (185/345)	46.4% (160/345)		
2007	Marani	30.1% (1145/3800)	69.9% (2655/3800)	119.45	< *0.0001*
	Kombewa	36.3% (340/937)	63.7% (597/937)		
	Iguhu	21% (984/4492)	79% (3508/4492)		
2003	Marani	95.4% (207/217)	4.6% (10/217)	1.78	0.1821
	Kombewa	93.9% (170/181)	6.1% (11/181)		
	Iguhu	96.2% (607/631)	3.8% (24/631)		

### Historical and current pattern of hospital anti-malarial prescriptions

**Fig. 8:**
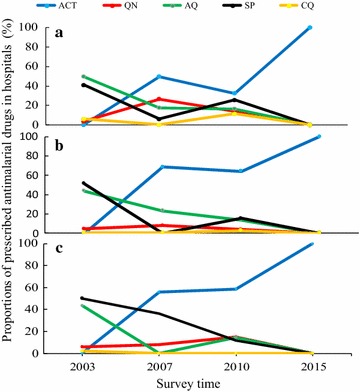
Prescription pattern of anti-malarial drugs in three hospitals in western Kenya from 2003 to 2015. **a** Iguhu Hospital, **b** Marani Hospital, **c** Kombewa Hospital

The anti-malarial outpatient prescriptions in three hospitals located in the study areas in 2003 included amodiaquine by 47% (487/1036), SP by 44.5% (461/1036), chloroquine by 4.2% (43/1036) and 4.3% (45/1036) quinine. Three years after malaria treatment policy change (year 2007), ACT covered most of the outpatient anti-malarial prescriptions by 60% (3786/6368) followed by amodiaquine (18% (1127/6368), quinine (15% (961/6368) and SP (8% (488/6368). By 2010 the compliance of ACT prescription dropped to 49.7% (412/829) of all prescriptions meanwhile SP rebound to 18.7% (155/829). ACT prescription patterns of Marani and Iguhu hospitals both declined between 2007 and 2010 but Kombewa Hospital had an improved compliance to the new drug policy. By 2015 all hospitals prescribed ACT by 100% (4042/4042) to all outpatient malaria confirmed cases (Fig. 8).

### Availability of anti-malarial drugs in the community drug outlets

**Fig. 9:**
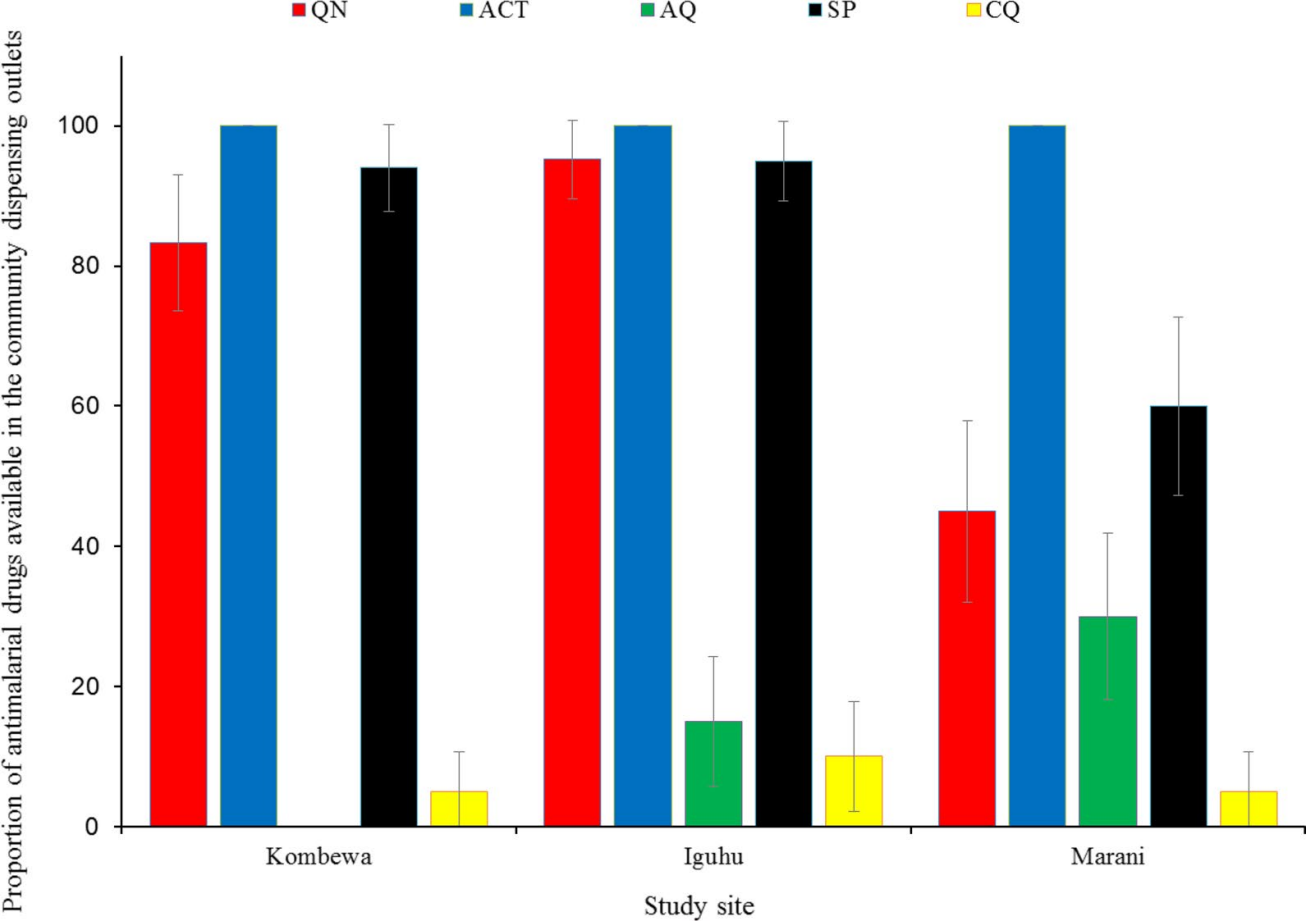
Availability of various anti-malarial drugs from the community drug dispensing outlets of three study sites with different responses of malaria interventions in western Kenya, 2015. *QN* quinine, *ACT* artemisinin-based combination therapy, *AQ* amodiaquine, *SP* sulfadoxine–pyrimethamine, *CQ* chloroquine

A survey of 59 drug selling outlets was made in 2015 at Kombewa (18/59), Marani (20/59) and Iguhu (21/59). Availability of ACT was 100% (59/59) followed by 81.4% (48/59) of SP and the injectable quinine found in 64.4% (38/59) all of drug shops. Other drugs were oral quinine (62.7% (37/59), amodiaquine (15.3% (9/59) and chloroquine (6.8% (4/59). However, availability of SP monotherapy drugs was highest at Kombewa (94.4% (17/18) followed by Iguhu (90.5% (19/21) while with 60% (12/20) of the selling outlets at Marani [exact = 7.99; p < 0.05] (Fig. 9).

### Precipitation and air temperature variability in western Kenya

**Fig. 10:**
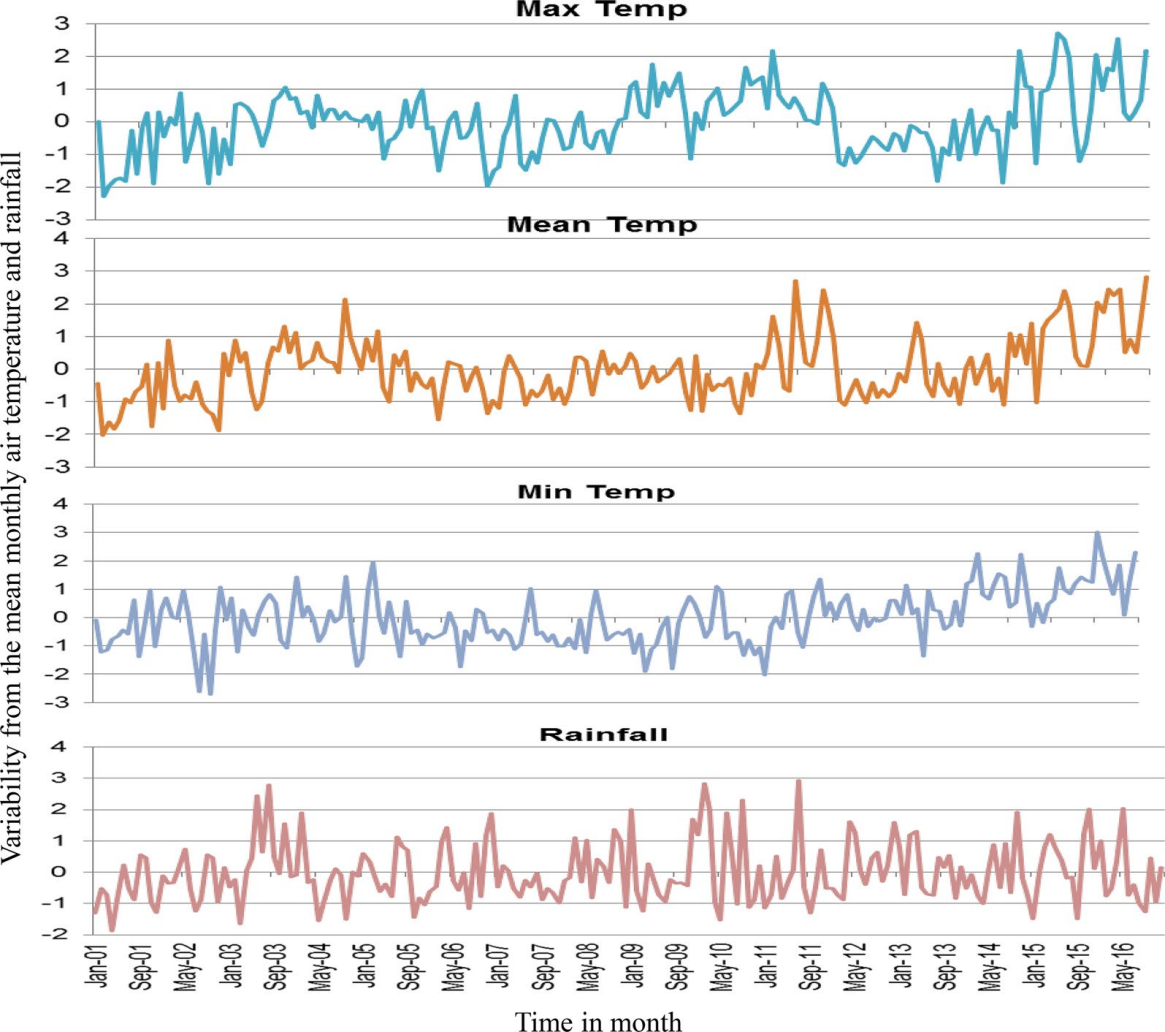
Long-term variability of air temperature and precipitation at Iguhu (Kakamega County) in western Kenya

**Fig. 11:**
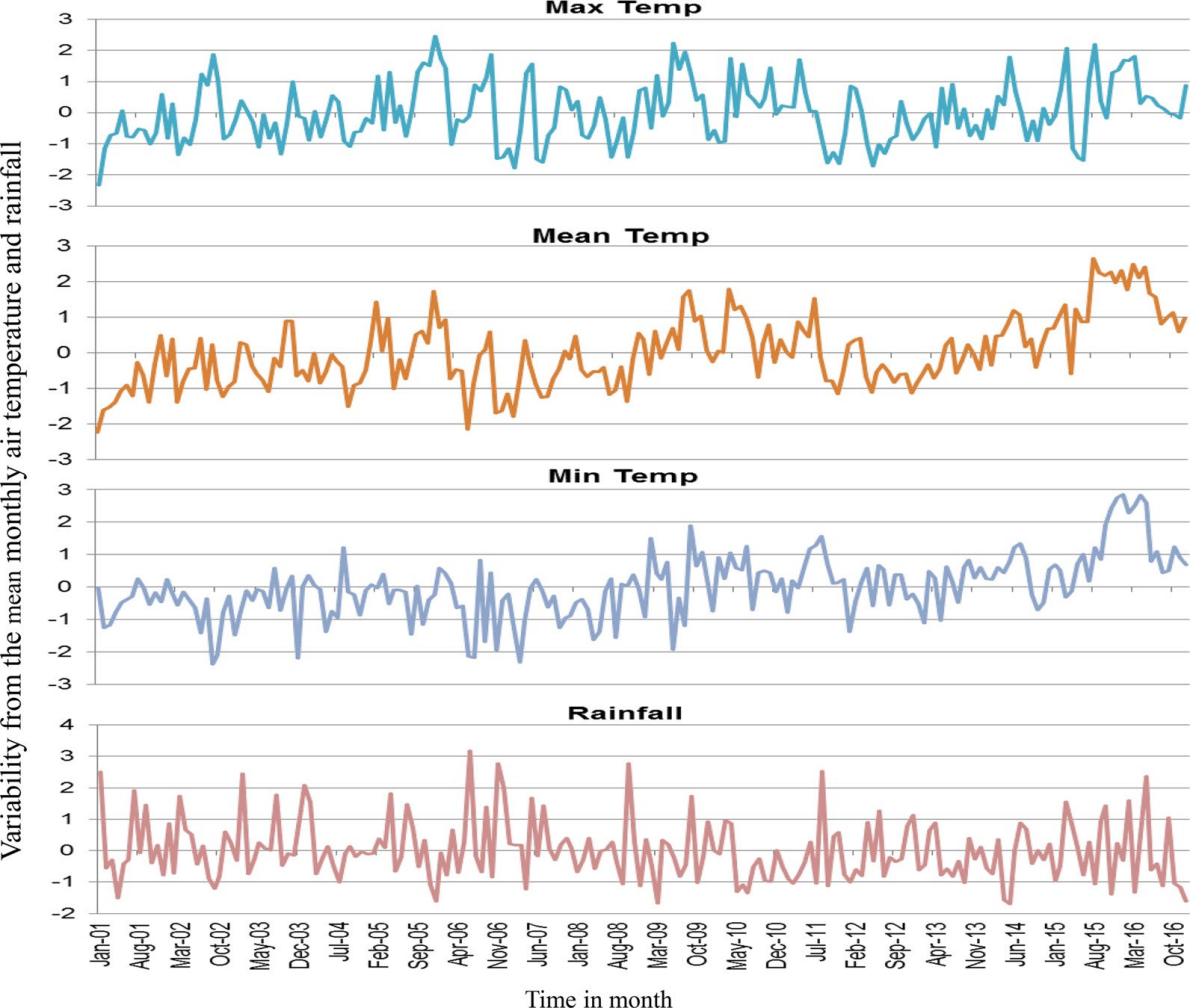
Long-term variability of air temperature and precipitation at Kombewa (Kisumu County) in western Kenya

**Fig. 12:**
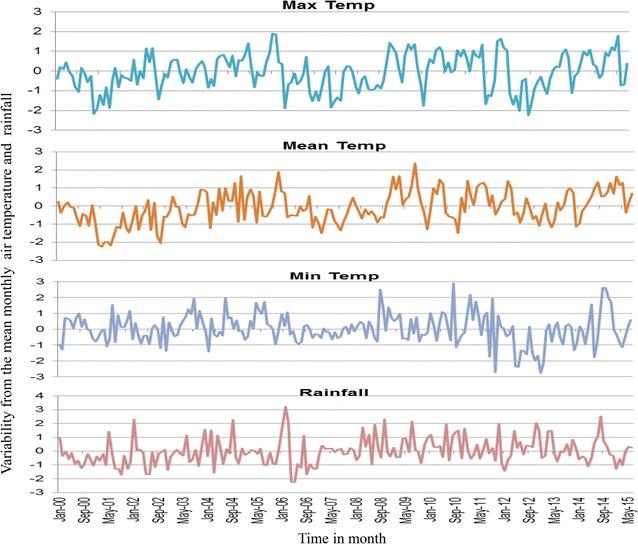
Long-term variability of air temperature and precipitation at Marani (Kisii County) in western Kenya

All sites showed increasing variations from the monthly means of maximum and minimum air temperatures as from 2011 to 2015. All study sites registered highest variation from the mean minimum temperatures in the recent years. Kombewa observed up to 2.8 °C in March 2016 and Marani up to 2.5 °C in September 2014 whereas Iguhu noticed the highest increase of 3 °C. Between 2013 and 2016 there was an increase of mean temperature by 2 °C for Marani and Iguhu which consequently led to increment of the mean of the minimum temperature at Marani to 16.13 °C in 2016. Marani also experienced highest rainfall increase in September 2014 (Figs. 10, 11, 12).

## Discussion

Sub-Saharan Africa still carry the highest malaria disease burden with 92% of the global mortalities [1]. However, there is a significant reduction of death by 40% between year 2000 and 2015 [2]. Similar success has been also observed in Kenya where the asymptomatic infections have been halved and significant decrease of clinical outpatient consultations as well as admissions [3, 4, 7]. The global malaria action plan (GMAP) of 2008–2015 targeted to reduce malaria cases by 75% but the infection in some areas in western Kenya was still increasing (Fig. 2). Existence of disease resurgence despite of the intensified interventions in the recent years has not only being seen in western Kenya but also in other countries of sub-Saharan Africa but with inadequate explanation on the cause [13, 15, 27].

This study found a significant reduction of indoor resting malaria vectors since the start of interventions in some sites (Fig. 4). Marani experienced an increase of indoor resting vectors from 2014 to 2016 which was also along with an escalation of asymptomatic parasitemia among primary school pupils (Figs. 2, 4). In conjunction with increased parasitemia at Marani, *An. funestus* s.l. became the main vector as they constituted three quarters of the total indoor female vectors and the rest being *Anopheles arabiensis*. Before interventions *An. gambiae* s.s. covered about 80% of all indoor resting vectors thereafter *An. funestus* s.l. took over. Elsewhere in East Africa studies have reported an increasing importance of *An. funestus* s.l. in malaria transmissions with an increase of not only abundance but also sporozoites rates [28, 29]. A study in western Kenya showed consistent findings as *An. funestus* s.l. was also found to have a raising abundance with highest sporozoite rate as compared to other vectors [30]. The LLINs ownership and use scale-up also changed the composition of malaria vectors at Kombewa, the population of *An. gambiae* s.s. reduced by 22% while that of *An. funestus* only decreased by 6%. Iguhu has the highest composition of *An. gambiae* s.s. with the least populations of *An. funestus* s.l. where there was a sustained low transmission and controlled vector population (Figs. 2, 4). One factor that clearly differentiates these areas is that sites with non-improving outcome of interventions (Marani and Kombewa) have *An. funestus* s.l. as the major vector (Fig. 5). The vector have shown to be the main malaria transmitting agent as a result of an increased sporozoites rates as well as abundance while exhibiting highest insecticide resistance to the widely used pyrethroids [30, 31]. Studies on insecticide susceptibility of this vector in East and South Africa found very high resistance to both deltamethrin and permethrin [29–32]. Populations of *An. funestus* s.l. from Kisii showed similar susceptibility upon deltamethrin exposure. Other studies in western Kenya also found as low as 10% mortality upon exposure to deltamethrin [30, 31]. The sustained control of malaria infection at Iguhu could be due to presence of insecticide susceptible *An. gambiae* s.s. as the major malaria vector (Figs. 5, 6). Whereas as Kombewa had a composition of all insecticide resistant vectors (*An. gambiae* s.s. as well as *An. funestus* s.l.) which could have limited the benefits of LLINs and IRS. Generally, study areas with high composition of insecticide resistant vectors experienced infection resurgence or sustained high transmission which is contrary to the global technical strategy for malaria 2016–2030 [1]. Moreover, having higher composition of highly anthropophilic *An. funestus* s.l. with such high resistance levels amid of changed biting behaviour increase chances of more malaria transmission [17, 30, 31, 33]. Nevertheless, the change in biting time from midnight to earlier or late has been reported from all the three study sites and, therefore, this could contribute to even more infection transmission potentials to areas with increasing or sustained high indoor vector densities [17, 33, 34].

Precipitations and air temperature among others factors significantly affect the breeding and population growth of malaria vectors [35, 36]. In East Africa highlands for example, climatic warming has been associated with malaria epidemics [37, 38]. The increasing monthly mean minimum and maximum ambient temperature from 2012 to 2015 was seen to all study sites. In conjunction with this, highest peak of rainfall at Marani was noted in September 2014. The combination of increased rainfall and air temperature increase at Marani gives the possible explanation of malaria resurgence in this area (Figs. 2, 4, 12). The increase of mean minimum temperature plays a major role on mosquito breeding in cool highland areas such as that of Kisii (mean annual temperature of 21.13 °C in 2016) than lowland warm areas [37]. The raise of vector populations at Marani was preceded by an increase of the mean minimum air temperature by 2.5 °C and rainfall (Figs. 2, 12). The climatic warming at Marani has resulted to an increase of the mean minimum temperature to 16.16 °C which might shorten the larvae stage and also gametocyte cycles in adult mosquitoes [38]. Along with this, increasing land use as a results of population growth have also contributed the increased suitable breeding sites and survivorship of *An. funestus* populations as it has been reported elsewhere [39].

The efficacy of ACT in western Kenya remains high despite of the reported increase of polymorphisms of specific key codons [40–42]. Availability of SP in drug dispensing outlets remains high but the use of this drug remains low (Table 2 and Fig. 9). Areas experiencing infection resurgence had lower use of SP and therefore drug resistance looks unlikely to explain the incident. The continued use of SP for presumptive treatment and self-prescriptions is consistent to the sustained SP specific codons polymorphisms while those of chloroquine diminishes [41, 43, 44]. Elsewhere in Africa, the sustained use of already resistant anti-malarials was associated with persistent malaria high transmissions [45]. Moreover, poverty has been associated with malaria morbidity and mortality for long but these three communities in western Kenya have similar social economic status and economic inequalities [46, 47].

The school-based surveillance of asymptomatic malaria demonstrates to be a better metric for monitoring transmission intensity and intervention effect size than the hospital based (Figs. 2, 3). For example at Kombewa, the number of positive cases in 2011 among primary school aged children was the same as in 2015 but the hospital survey shows higher number of cases in 2011 than 2015 (Figs. 2, 3). This could be due the fact that the later surveillance system may be affected by number of factors like case management rate, reporting rate and case confirmation. In western Kenya, malaria diagnosis and treatment used to be based on blood slide as well as clinical judgment, therefore some cases in 2011 could be clinically diagnosed and reported as confirmed [48]. However, malaria case detection following the introduction of rapid diagnostic tests and blood slide microscopy training over the recent years has greatly improved [49]. Moreover, the hospital-based surveillance system is often interrupted by frequent strike of physicians and nurses. Asymptomatic malaria prevalence data among school age children was collected systematically and thus more reliable than hospital-based malaria case data (Figs. 2, 3). This study also found of an increased LLINs ownership to over 80% in all areas, studies however shows lowest use among the 5–14 age group (school age) [25]. The unpublished data from the study sites shows 72 and 58% of this age group slept under LLINs a night before survey at Marani and Kombewa, respectively. Whereas at Iguhu (areas showing sustained low transmission), only 50% of the 5–14 age group slept under LLINs. The use of LLINs among the school age was highest at Marani (malaria resurgence site) and lowest at Iguhu (an area with controlled transmission). One would expect to see highest use of LLINs in an area that has attained sustained transmission control but the opposite is true. This means that other factors like increase in vector population and insecticide resistance could be the likely major drive of infection transmissions in these populations. The over 80% LLINs coverage could have provided community wide protective effect [50] to all study sites despite of low use among the school age but the explaining reasons for the observed variation in response to interventions are likely to be type of the vector, population density and insecticide susceptibility (Table 1). In western Kenya, suitability of asymptomatic malaria surveillance in schools has been evaluated and found to be representative of the general population [51]. Therefore, the described trend of malaria transmission which also correlates with the indoor vectors populations represents the true infection transmission dynamics in the study area (Figs. 2, 4). This study however lacks information of the long-term malaria case management rates, site specific *An. funestus* s.l. insecticide susceptibility and site specific vector behavior. This information would have improved the analysis on the cause of the observed changing dynamics of malaria infection in western Kenya.

## Conclusions

The sustained highest composition of the highly anthropophilic *An. funestus* s.l. and also availability of pyrethroids resistant *An. gambiae* s.l. could be the cause of the sustained high malaria transmission at Kombewa. The increase of the mean minimum air temperature and precipitation have led to an increased abundance of insecticide resistant *An. funestus* s.l. population at Marani which may have subsequently caused the observed infection resurgence. At Iguhu where there was a sustained control of infection, the pyrethroids moderately resistant *An. gambiae* s.s. had the highest composition. Climate variability, insecticide resistance and vector population shift are likely the cause of the contrasting outcome of malaria interventions n western Kenya. To meet the GMAP 2030 targets there is a call for use of carbamates and organophosphates for indoor targeted interventions and expansion the integrated vector management [16, 31]. Along with this, the continued surveillance of vectors and clinical and subclinical infection is highly recommended for changing infection transmission risks. Monitoring of insecticides resistance should be done along with use of air temperature and precipitation data to predict vector and parasite dynamics especially in highlands where slight changes in these parameters could lead to devastating infection outbreaks. Malaria transmission competence and biting behaviour of re-emerging *Anopheles funestus* complex should be also studied.
